# Quad-Port Circularly Polarized MIMO Antenna with Wide Axial Ratio

**DOI:** 10.3390/s22207972

**Published:** 2022-10-19

**Authors:** Vamshi Kollipara, Samineni Peddakrishna

**Affiliations:** School of Electronics Engineering, VIT-AP University, Amaravati 522237, India

**Keywords:** antenna, axial ratio (AR), circular polarization (CP), characteristic mode analysis (CMA), MIMO, quad port

## Abstract

This article studies a quad-port multi-input-multi-output (MIMO) circularly polarized antenna with good isolation properties. Using characteristic mode analysis (CMA), the first six distinct modes of the asymmetric square slot with an inverted L-strip are analyzed. In this study, modal parameter extraction is carried out for circular polarization (CP) radiation. A simple annular ring microstrip feed is excited to obtain broadband CP based on CMA. The single-unit feeding structure is replicated orthogonally four times to achieve a CP MIMO antenna. This antenna provides port isolation of more than 21 dB without the use of an additional decoupling element. The quad-port CP-MIMO antenna is simulated with a total dimension of 50 × 50 mm^2^. The antenna attains impedance matching (S_11_ < −10 dB) from 5.37 GHz to beyond 11 GHz with an axial ratio bandwidth (ARBW) of 4.65 GHz (5.61 GHz to 10.26 GHz). The peak realized gain of the MIMO antenna is measured at 5.69 dBi at 8.4 GHz. Additionally, the diversity performance parameters of the MIMO structure are computed. The advantages of the proposed structure have been evaluated by comparing it to previously reported MIMO structures. A prototype of the MIMO structure measurements was found to match the simulation results.

## 1. Introduction

The rapid advancement of wireless communications services is attributed to the increasing demand for higher transmission rates and significant transmission capacity. However, it has become apparent that the limited frequency spectrum is the primary barrier to the advancement of wireless communication. To make out of the confined frequency spectrum resource overhead, the multiple-input multiple-output (MIMO) technology has been developed and intensively investigated [1,2]. The MIMO antenna has the potential to significantly increase channel capacity and thus improve spectral efficiency and reliability [3]. In the design of a MIMO antenna, the four most important parameters such as space, operating bandwidth (BW), polarization, and mutual coupling are considered.

When designing the MIMO antenna, the antenna elements should fit within the limited space available. This is a very difficult task for a practical communication system. Because of the limited space, the antenna elements are tightly packed, so that the elements are strongly coupled together. In addition, there is a potential for interference between two antenna elements, especially around feeding ports, due to their location on the same ground plane [4]. To address this issue, many studies have concentrated on decoupling methods [5,6,7,8,9,10,11,12,13,14,15,16,17,18]. The insertion of different slits into the ground plane is the easiest way to suppress the coupling [5,6,7]. Alternatively, parasitic elements [8,9] can be placed between or in proximity to MIMO elements. Here, feeding techniques or the use of multiple-layer methods increase the complexity. In addition, the use of a defective ground (*DG*) [10] UWB slot antenna was proposed. However, the structure comprises only two element MIMO antennas. In addition, metamaterials [11,12,13], electromagnetic bandgap (EBG) [14,15,16], and frequency selective surfaces (FSS) [17,18] could also be an effective alternative. The use of complex metamaterial or EBG renders it unsuited for highly sophisticated systems. The FSS as a single layer with air space increases the profile of the antenna. In other structures, the authors addressed the above issues by proposing a four-element ultra-wideband [19] and quadband [20] MIMO antenna. At the same time, these antennas are isolated over 17 dB and 22 dB between the adjacent elements. These antennas [5,6,7,8,9,10,11,12,13,14,15,16,17,18,19,20] can only do the linear polarization operation.

On the other hand, circularly polarized antennas are more effective at reducing polarization mismatch and blocking multipath interference [21]. In the current scenario, the implementation of the circular polarization (CP) multi-port antenna is a challenging task. It becomes very difficult to maintain orthogonal degenerate modes with a quarter-phase difference while maintaining isolation [22]. Over the past few years, several CP antennas have received more attention for use in MIMO antennas with mutual coupling reduction [23,24,25,26,27]. It was observed that the design contains a 2 × 2 array MIMO antenna [23] that achieves isolation of more than 33 dB between the ports with CP. However, this structure is only intended for narrowband applications. In an alternative approach, a 2 × 1 wideband antenna with a mutual coupling of less than −25 dB [24] has been proposed. This antenna was realized with an edge-to-edge spacing of 0.3λ with low realized gain at their operating frequency. A compact MIMO antenna for frequencies below 6 GHz that was isolated at 15 dB is also proposed, but its gain was relatively small [25]. Ullah et al. [26] proposed a compact CP MIMO antenna with polarization diversity with isolation of more than 20 dB. A coplanar asymmetric T-shaped feed square slot antenna with an inverted L strip ground plane was proposed for wide ARBW [27] with isolation of more than 15 dB. The MIMO antennas [26,27] provide dual CP but have a very small axial ratio(AR) and impedance bandwidth (IPBW).

On the other hand, a two-port dielectric resonator antenna (DRA) was proposed with a hybrid technique to reduce mutual coupling [28]. Here, with a parasitic patch and diagonally positioned radiating elements, mutual coupling is suppressed to less than −28 dB for worldwide interoperability for microwave access (WiMAX) applications. A two-port DRA without any separation between the elements achieves the isolation of more than 15 dB for C band applications [29]. Considering the operational frequency of these models, the overall size is relatively large. In another design, with a traditional cross slot to accomplish the response of CP with an isolation of 11 dB [30] was investigated. In addition, to eliminate coupling, a complex multi-layer design has been proposed at the millimetric wave frequency using the FSS with an isolation of more than 10 dB [31]. Moreover, the literature presented in [23,24,25,26,27,28,29,30,31] is restricted to designs with two ports, which is another limitation. Alternatively, in [32], a three-port pattern and polarization diversity antenna were proposed with isolation greater than 15 dB.

To enhance simultaneous data streams, different researchers focus on designs with four port antennas with reduced mutual coupling [33,34,35]. A diagonal isolating network is introduced between neighboring radiating elements to provide high isolation of approximately 25 dB [33]. In spite of its wide IPBW, this antenna has a very narrow AR bandwidth (ARBW). Kumar et al. [34] proposed wide ARBW with four G-shaped monopole elements for dual CP. It attains isolation of more than 18 dB for C-band applications with an overall size of 70 × 68 × 1.6 mm^3^. In another design, a truncated hexagonal CP antenna has been proposed [35] to simultaneously improve ARBW and IPBW where isolation was more than 18 dB. Here, the decoupling network is designed by introducing additional metal strips with 180° out of phase. More recently, to design a precise decoupling network to achieve improved port isolation without affecting antenna performance, characteristic mode analysis (CMA) was introduced. Recent research has revealed the classical CMA theory with port isolation of greater than 15 dB for a triple-band dual-polarized MIMO antenna [36]. This antenna was designed at frequencies of 1.54 GHz, 2.5 GHz with linear polarization, and 5.63 GHz with CP. Understanding the information provided by the evolved structure is motivatating to enhance the performance in the wide ARBW applications. As a summary, the proposed literature is compared in Table 1 with their isolation techniques and polarization.

Unlike the above-mentioned MIMO structures, this work addresses a simple quad-port CP-MIMO antenna with CMA. To take the advantage of CMA, the antenna features can be extracted in an appropriate way. In addition, it highlights the lack of physical understanding in the development of the basic antenna design before any feed is applied for excitation [37]. The CMA gives details about antenna resonances that can be predicted by extracting modal parameters. These parameters can be considered to be modal significance (MS), and characteristic angle (CA). Based on MS and CA, a simple coplanar waveguide slot antenna design is proposed for examining CP radiation. More precisely, the MS and CA help to generate CP radiation [38,39]. These parameters make it possible to optimize the IPBW and ARBW. Further, based on the single-slot antenna, the design is extended to a four-port MIMO configuration without impacting the parameters of an individual element.

The four-port CP-MIMO provides IPBW (S_11_ < −10 dB) of >65% (5.37 GHz to beyond 11 GHz) and ARBW (<3 dB) of 58.6% (5.61 GHz to 10.26 GHz). The measured isolation between the ports is observed to be greater than 15 dB. The envelope correlation coefficient (ECC) and diversity gain (*DG*) are observed below 0.003 and 10 dB, respectively. The total active reflection coefficient (*TARC*) (0.0001 dB) and mean effective gain (*MEG*) (−3 dB) within the band are extracted. The antenna supports C-band uplink and X-band(military) both uplink and downlink applications(military). In addition, the diversity parameters also show little dependence on the excitation phase in the design. The computer simulation tool is used to generate all simulation results throughout the overall design process. The organization of the paper is divided into the following sections: Section 2 presents the design and evaluation of a four-port MIMO antenna. This section covers the evaluation of CP radiation using CMA followed by a four-port antenna design procedure. Section 3 presents the results and discussion associated with various simulated and measured parameters, followed by a comparison of the related literature. Section 4 deals with the conclusion of the proposed design.

## 2. Design and Evolution of Four-Port MIMO Antenna

### 2.1. Evolution of CP-Radiation Using CMA

**Figure 1:**
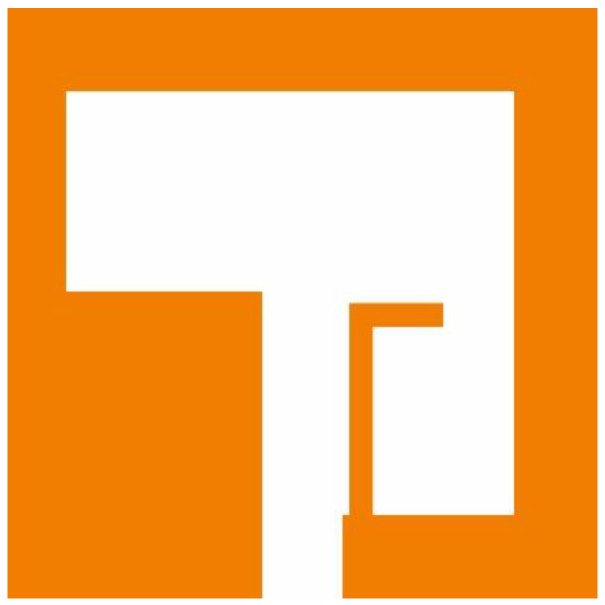
Evolution of single-port antenna for characteristic mode analysis (CMA).

**Figure 2:**
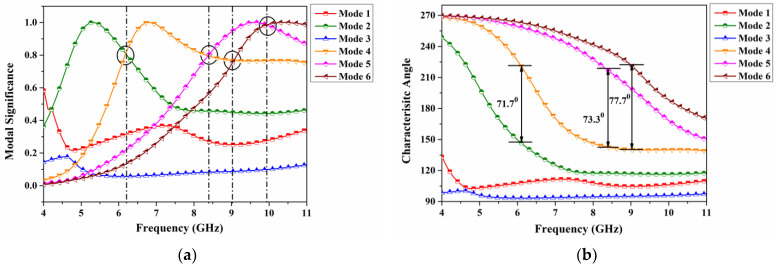
Modal parameters: (**a**) Modal significance and (**b**) Characteristic angle.

This section describes the evolution of the modal behavior for employing CP behavior without feeding into the design, as shown in Figure 1. To achieve the designated structure shown in Figure 1, various other sequential steps have been analyzed. Those are not represented here for the sake of brevity. The CMA is analyzed for the first six modes as shown in Figure 2. From the MS parameter, significant orthogonal modes such as modes 2, 4, 5, and 6 are observed at 5.32 GHz, 6.80 GHz, 9.61 GHz, and 10.40 GHz, respectively. In addition, the MS indicates that modes 1 and 3 are no longer significant within the specified band. Broader frequency bandwidth is achieved through significant modes. Furthermore, the intersection point of the significant modes offers to analyze the CP behavior of the antenna. From Figure 2, it is observed at 6.19 GHz, 8.4 GHz, and 9.9 GHz between mode 2 and mode 4, mode 4 and mode 5, and mode 5 and mode 6, respectively. The MS value at the above three frequencies is reported as 0.81, 0.98, and 0.76. Moreover, the CA observed simultaneously at these frequencies are 71.7°, 73.3°, and 77.7°, respectively. From all the intersecting points except mode 5 and mode 6, the CA stabilizes to more than 70°, which contributes to CP radiation by providing a proper feeding in the design with an additional phase shift. Table 2 summarizes the modal parameters.

**Figure 3:**
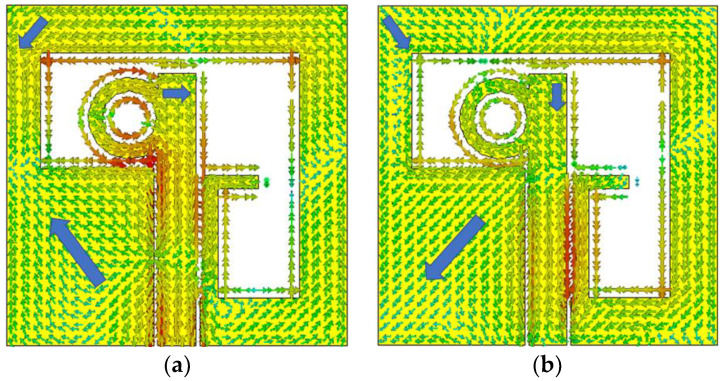

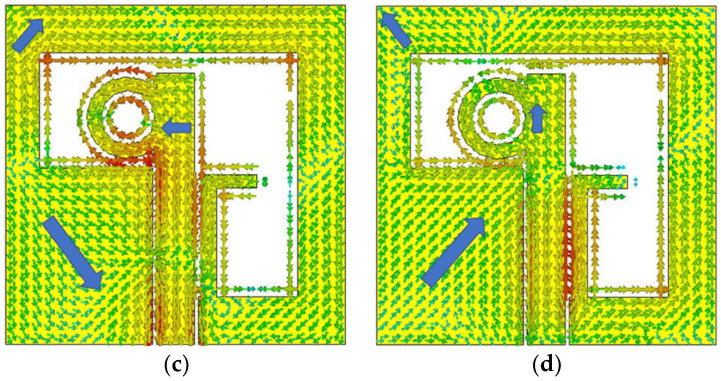
Surface current distribution of proposed antenna at 7.5 GHz. (**a**) 0° (**b**) 90° (**c**) 180° (**d**) 270°.

Based on the above analysis, it is observed that all three orthogonal modes are appropriate for CP by considering the additional phase shift from the feed network. To achieve the additional phase shift, the parametric variation of the feed length is carried out by excitation from the bottom center of the slot. By varying the feed length, the AR is observed around 6 GHz, with narrow ARBW. In the next step to improve the ARBW, an annular ring is attached to the feeder structure. Because of this annular ring, the extra phase shift is intended for other higher-order modes that generate the wide ARBW. To understand the CP behavior, the surface current distribution analysis is analyzed at some arbitrary frequency (7.5 GHz) within the band. The surface current analysis with different phase instances is shown in Figure 3. It can be shown that surface current has similar magnitudes, but quadrature is in phase, indicating that CP attributes are predicted. As shown, the top left of the asymmetric patch rotates counterclockwise (CCW) with a phase difference of 90° at all phases. In addition, there is a 90°-phase differential in all phase variations from 0° to 270° at these positions. Moreover, it is also observed that the current is distributed clockwise (CW) at the point of intersection of the upper edge between the annular ring and the feed line. With CCW rotation on the asymmetric slot patch and CW rotation on the feedline, it is concluded the antenna presents left-hand CP. Further, to investigate the MIMO antenna, the single CP antenna is comprised of four-identical antennas located orthogonally to each other. The analysis and design process is presented in the following sections.

### 2.2. Quad-Port Antenna Design

**Figure 4:**
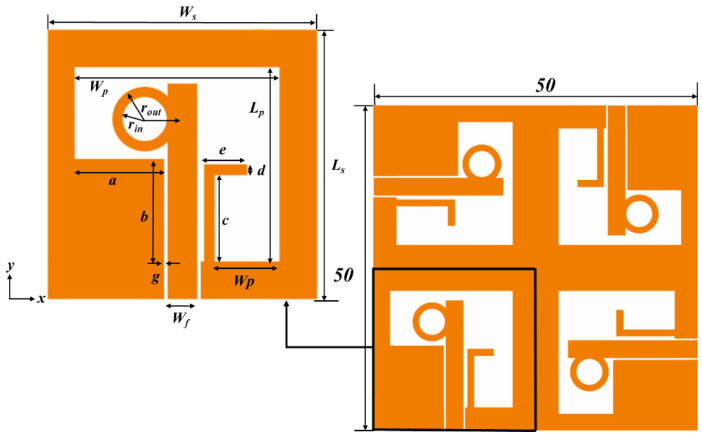
Four-port antenna design.

Figure 4 illustrates the structure of an annular ring microstrip feed quad-port CP MIMO antenna. It occupies the dimensions of 50 × 50 mm^2^. It is designed on a single-layer FR-4 substrate with a 0.4 mm thickness. Of the four ports, a single-element design consists of an asymmetrical square slot with an inverted L-strip. The annular ring is attached to the feed line to achieve CP radiation. The designed single port overall dimensions are depicted in Table 3.

**Figure 5:**
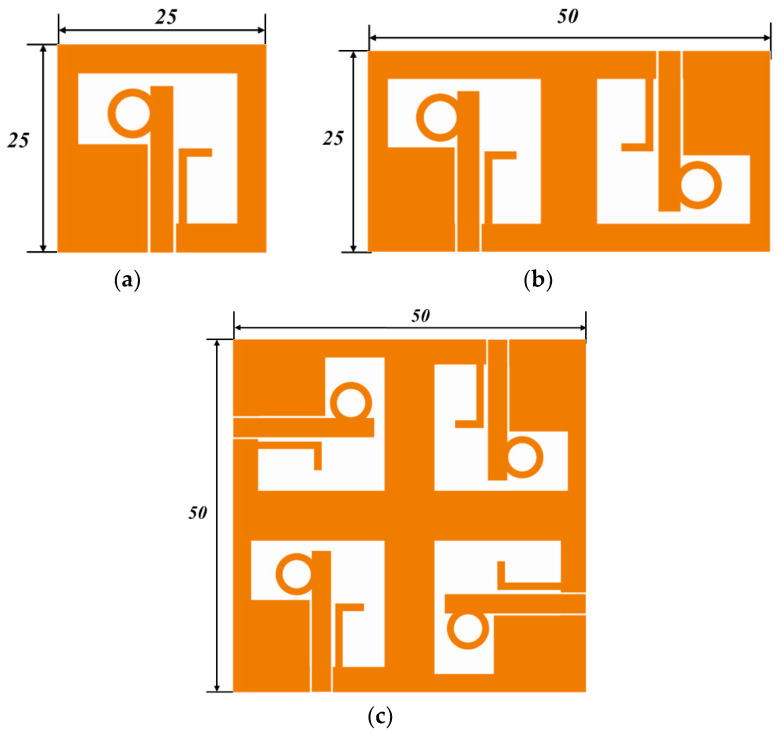
Design process of the quad-port CP-MIMO antenna (**a**) Ant @ 1, (**b**) Ant @ 2, and (**c**) Ant @ 3.

**Figure 6:**
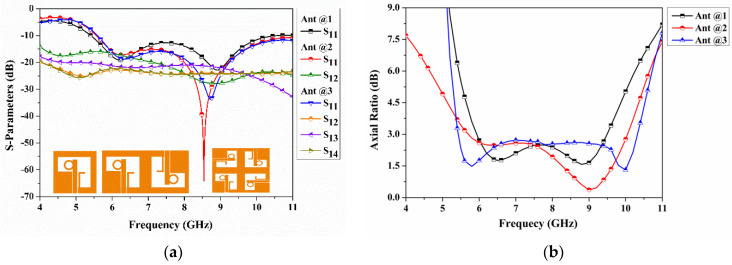
Scattering and axial ratio results for Ant @ 1, Ant @ 2, and Ant @ 3 (**a**) S-Parameters and (**b**) Axial ratio.

The design process of the quad-port CP-MIMO antenna from a single port is described in three different stages, as shown in Figure 5. In the first stage, a single port CP antenna is designed with 25 × 25 mm^2^, as illustrated in Figure 5a. In the second stage, the single port antenna is mirrored with an 180° phase shift, as shown in Figure 5b. It occupies a size of 50 × 25 mm^2^. At the final stage, it is reproduced on 50 × 50 mm^2^ dimensions with a quadrature phase, as depicted in Figure 5c. These three antennas from Figure 5a–c are denoted as Ant @ 1, to Ant @ 3, respectively. Their simulated S_11_ and AR are shown in Figure 6. The Ant @ 1 offers an IPBW < −10 dB from 5.41 GHz to 10.54 GHz, and ARBW from 5.92 GHz to 9.50 GHz. While the IPBW and ARBW are observed for Ant @2 from 5.56 GHz to beyond 11 GHz and 5.69 GHz to 10.05 GHz, respectively. The final stage antenna named Ant @3 provides IPBW beyond 11 GHz from 5.59 GHz and ARBW from 5.54 GHz to 10.31 GHz. Additionally, a slight deviation of the ARBW from the broadside direction at higher frequencies was observed. The design provides isolation of more than 18 dB without employing special isolation improvement techniques. Additionally, it is noticed that the realized peak gains (simulated) of single-port, dual-port, and quad-port antennas are 3.66 dBi, 4.55 dBi, and 5.48 dBi, respectively. The comparative analysis of the foregoing discussion is summarized in Table 4.

## 3. Result and Discussion

**Figure 7:**
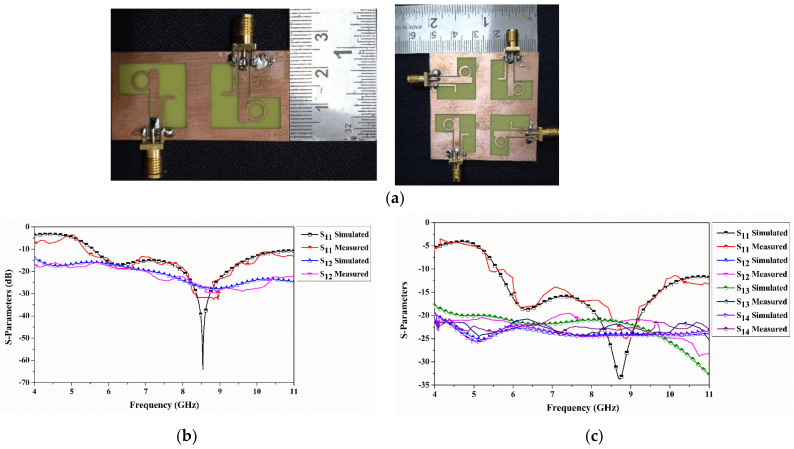
(**a**) Antenna prototype, (**b**) Dual-port S-parameters, and (**c**) Quad-port S-parameters.

Figure 7a shows the fabricated dual- and four-port CP-MIMO antenna. The simulated S-parameters of dual and quad port results are validated with measured results using a vector network analyzer. Figure 7b,c shows the S parameter comparison of dual- and quad-port MIMO antenna, respectively. The measured reflection parameters of dual-port antenna are seen from 5.40 GHz to over 11 GHz. This will result in a deviation of 0.16 GHz in the lower spectrum bands. The transmission parameter (S_12_) isolates more than 21 dB and is observed in simulation at more than 18 dB at 7.1 GHz. Similarly, the quad-port antenna measured |S_11_| results are seen beyond 11 GHz from 5.37 GHz. This result in a deviation of 0.22 GHz to a lower frequency. In addition, it can be seen that the isolation difference between simulation to measurement is greater than 3 dB. In the simulation, it is noted as more than 18 dB and when measured as 21 dB. This will indicate the antenna provides better performance in the MIMO system. Furthermore, this antenna covers military X-band uplinks and downlinks as well as C-band downlinks applications.

**Figure 8:**
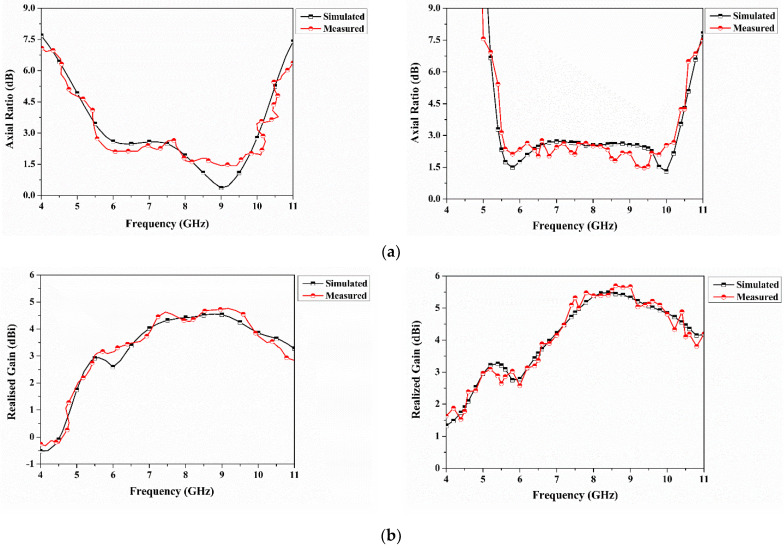
(**a**) Axial ratio of dual- and quad-port antenna and (**b**) Realized gain of dual- and quad-port antenna.

Figure 8 shows the comparison of the AR and gain measurements of the dual- and quad-port antenna with the simulation. The measured ARBW as shown in Figure 8a is observed at 5.55–10.01 GHz and 5.61–10.26 GHz for dual and qua ports, respectively, while the simulation ARBW is 5.69–10.05 GHz and 5.54–10.31 GHz. The simulated and measured minimum AR values for the quad-port antenna are observed at 1.31 dB (10 GHz) and 1.46 dB (9.4 GHz), respectively. In Figure 8b, the difference in gain achieved with the measurement values is observed at 0.21 dBi and 0.17 dBi for quad and dual ports. The peak measured gain is observed as 5.69 dBi and 4.72 dBi, respectively. The foregoing discussion is summarized in Table 5 and Table 6.

**Figure 9:**
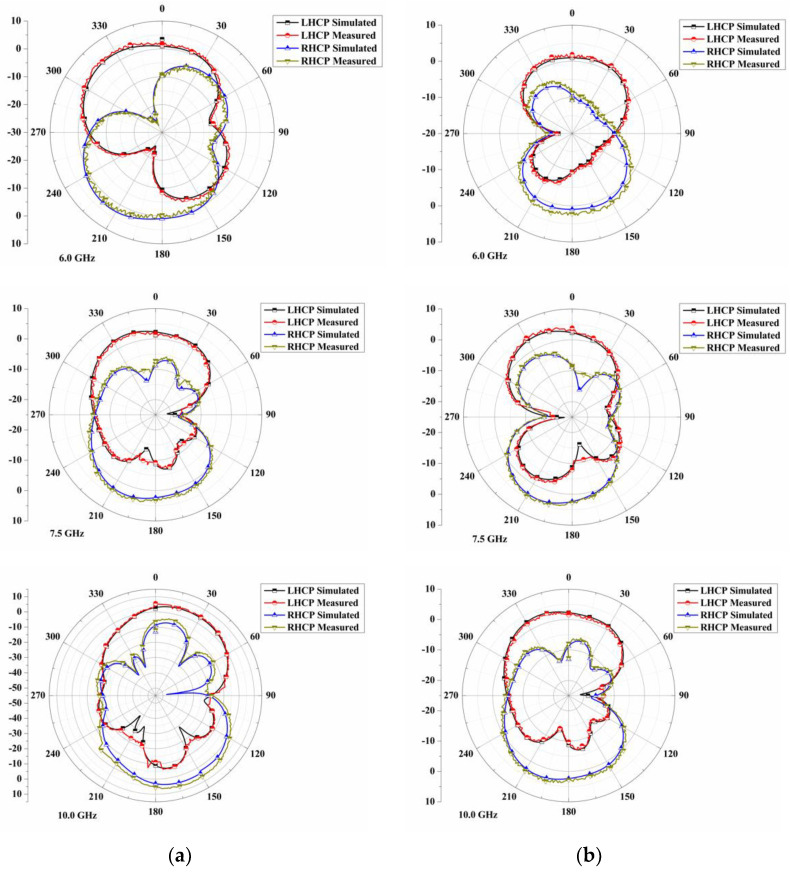
Radiation pattern comparison (**a**) x-z plane and (**b**) y-z plane.

The direction of CP in the far field is measured at 6.0 GHz, 7.5 GHz, and 10.0 GHz for the quad port antenna in the left-hand CP (LHCP) and right-hand CP (RHCP). Figure 9 illustrates the comparison of simulation and measurement patterns in the x-z (0°) and y-z (=90°) planes. Deviations in results are due to fabrication and measurement errors. According to the radiation patterns, the magnitude of both planes is identical to their respective frequencies. From the main lobe direction, the magnitude of the simulated LHCP in the x-z plane is observed to be 2.92 dBi, 3.12 dBi, and 4.28 dBi at 6.0 GHz, 7.5 GHz, and 10.0 GHz, respectively, whereas the magnitude of LHCP in the y-z plane is 1.15 dBi, 3.76 dBi, and 3.16 dBi in the direction of the main lobe, respectively.

Additionally, the magnitude difference between LHCP and RHCP in the x-z plane between 0° and 180° is 0.03 dBi, 0.06 dBi, and 0.1 dBi, respectively, for 6.0 GHz, 7.5 GHz, and 10.0 GHz. The magnitude difference in the y-z plane at the above frequencies is 0.01 dBi, 0.07 dBi, and 0.11 dBi, respectively. In all frequencies, the RHCP component is somewhat larger than the LHCP component, and the magnitude difference is within acceptable limits. A pattern at lower and medium frequencies is almost in boresight, whereas a pattern at higher frequencies is slightly off in the broadside direction.

Further, this article includes other surpassing parameters for the quad-port antenna in order to demonstrate its effectiveness regardless of the excitation phase. An essential parameter in this regard is the ECC. Essentially, it is a measurement of the correlation between the different antenna elements in a MIMO structure. An ECC is calculated from S-parameters or a far-field Equations (1) and (2). In the case of any two-element MIMO antenna, it is defined as follows [40,41]:

(1) $$\zeta_{ij}=\frac{|S_{ii}^{*}S_{ij}+S_{ji}^{*}S_{jj}{|}^{2}}{\left(1-(|S_{ii}{|}^{2}+|S_{ij}{|}^{2}))(1-(|S_{jj}{|}^{2}+|S_{ji}{|}^{2})\right)}$$

(2) $$\delta_{ij}=\frac{|\iint_{\;4\pi \vec{R_{i}}}(\theta ,\phi ){•}_{\vec{R_{j}}}{\left(\theta ,\phi )\right|}^{2}}{\iint_{\;4\pi }{|}_{\vec{R_{i}}}{\left(\theta ,\phi )\right|}^{2}\;d\Omega \iint_{\;\;4\pi }{|}_{\vec{R_{j}}}\left(\theta ,\phi \right){|}^{2}d\Omega }$$

where $\vec{R_{i}}(\theta ,\phi )$ refers to the radiation pattern when the *i*th port of the mn MIMO antenna system is energized. Additionally, the • symbol denotes the Hermitian product.

**Figure 10:**
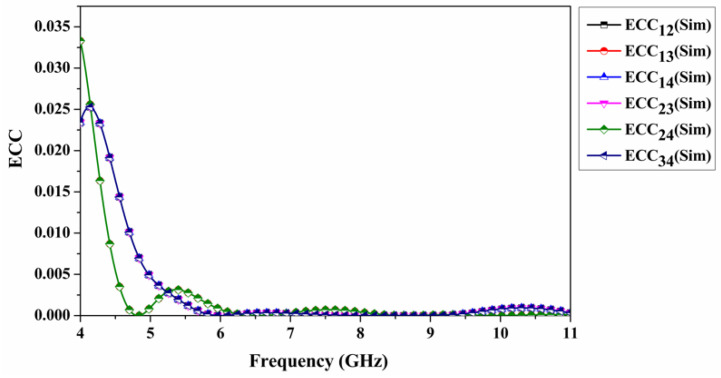
Simulated envelope correlation coefficient of quad-port antenna.

As shown in Figure 10, S-parameters are used to compute ECC in simulated environments. Ideally, the value should be less than 0.5. For all port combinations in the designated range (ECC_12_, ECC_13_, ECC_14_, ECC_23_, ECC_24_, and ECC_34_), the proposed four-port CP-MIMO antenna is less than 0.003. It is essential to maintain this low-value requirement to prevent the degrading of the signal-to-noise ratio under diverse circumstances. There is also another parameter involved in this condition called *DG*. This is the parameter used to detect multiple path signals that are transmitted by the same transmitter. Equation (3) is used to calculate it [42].

(3) $$DG=10\sqrt{1-|\zeta_{ij}{|}^{2}}$$

**Figure 11:**
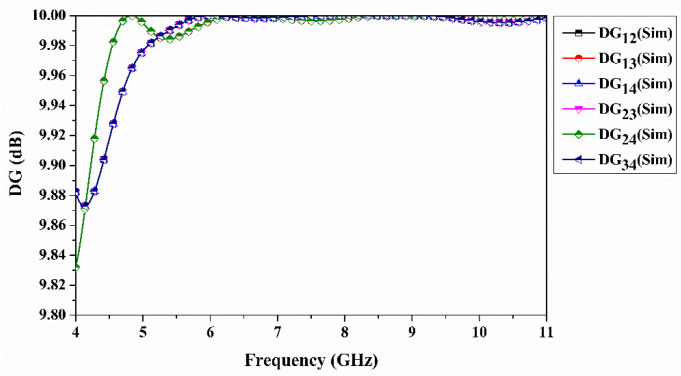
Simulated diversity gain of quad-port antenna.

For the proposed CP-MIMO antenna, *DG* is less than 10 dB for all cases, as shown in Figure 11. It demonstrates that the antenna performs adequately in terms of diversity. The above-mentioned parameters of the MIMO antenna are not taken into account with the impact of the input signal phase. From this, another key parameter called *TARC* should be taken into consideration. It measures the reflecting coefficient of the signal phases. *TARC* is calculated for a multiport antenna system using the following Equation (4) [43].

(4) $$\Gamma_{a}^{t}=\frac{\sqrt{\sum_{i=1}^{n}|bi{|}^{2}}}{\sqrt{\sum_{i=1}^{n}|ai{|}^{2}}}$$

where *b_i_* and *a_i_* are the reflected and incident signals, respectively. These are related to the *S* parameters matrix as

*b* = *Sa* (5)

where

*S* = [*s*11 *s*12] (6)

Considering a two-port MIMO antenna, *TARC* is evaluated as (7).

(7) $$TARC=\sqrt{\frac{{\left(S_{11}+S_{12}\right)}^{2}+{\left(S_{21}+S_{22}\right)}^{2}}{2}}$$

**Figure 12:**
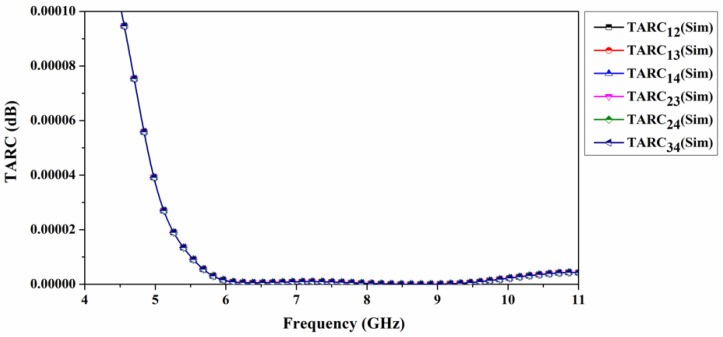
Simulated total active reflection coefficient of quad-port antenna.

The *TARC* values for the four-port antenna are shown in Figure 12 (i.e., *TARC*_12_, *TARC*_13_, *TARC*_14_, *TARC*_23_, *TARC*_24_, *TARC*_34_). When no waves are reflected from the port, the ideal expected *TARC* values should be 0 dB. However, in the suggested MIMO antenna, the simulated *TARC* values are 0.0001 dB for all possible combinations of ports.

Another important MIMO diversity parameter is called *MEG*, which quantifies the mutual interaction between antenna elements and the statistical properties of the propagation environment. The *MEG* is helpful in understanding the power imbalance from various parameters such as gain and total efficiency. *MEG* is the ratio of the MIMO antenna receiving power to the total power incident. It is calculated using Equation (8) [44].

(8) $$MEG_{m}=0.5\{1-\sum_{j=1}^{k}\left|S_{mn}{|}^{2}\right\}$$

where *k* = number of antennas, *I* = antenna under construction.

Therefore,

(9) $$MEG_{1}=0.5\{1-|S_{11}{|}^{2}-|S_{12}{|}^{2}-|S_{13}{|}^{2}-|S_{14}{|}^{2}\}$$

(10) $$MEG_{2}=0.5\{1-|S_{21}{|}^{2}-|S_{22}{|}^{2}-|S_{23}{|}^{2}-|S_{24}{|}^{2}\}$$

(11) $$MEG_{3}=0.5\{1-|S_{21}{|}^{2}-|S_{22}{|}^{2}-|S_{23}{|}^{2}-|S_{24}{|}^{2}\}$$

(12) $$MEG_{4}=0.5\{1-|S_{41}{|}^{2}-|S_{42}{|}^{2}-|S_{43}{|}^{2}-|S_{44}{|}^{2}\}$$

**Figure 13:**
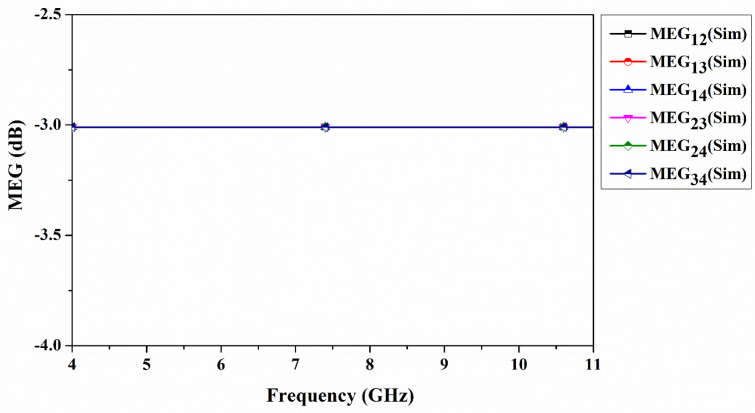
Mean effective gain of quad-port antenna.

The *MEG* difference of any two antenna elements should not be more than −3 dB. Figure 13 shows the *MEG_ij_* of the four-port antenna. This demonstrates the diversity performance of the proposed antenna. Finally, to show the advantage of the proposed design, Table 7 presents the comparison of the four-port CP-MIMO antenna with the MIMO antenna designs in the literature. The comparison includes size, number of ports, polarization type, IPBW, ARBW, and peak gain. In comparison with the literature except [34], the proposed antenna provides wide ARBW. Compared to the proposed structure, this structure is relatively large. 

## 4. Conclusions

A simple quad-port CP-MIMO antenna is fabricated, characterized, and compared to its simulation results. A single antenna element is arranged orthogonally across 360° to form four identical antenna elements for quad port MIMO. The proposed CP-MIMO antenna design provides an IPBW > 64.9% and ARBW of 58.6%. The antenna covers both the C-band uplink and X-band uplink and downlink applications. The CP-MIMO antenna maintains isolation greater than 21 dB in the operating band. The final fabricated antenna achieves a gain of 5.69 dBi. It is obvious that the antenna presented is well represented in terms of diversity (ECC < 0.001, *DG* 10 dB, *TARC* 0.0001 dB, *MEG* −3 dB), insulation, impedance correspondence, and AR. From a future perspective, the CMA approach to antenna design becomes attractive if CP antennas can satisfy the requirements of long-distance communication as done by high gain linearly polarised antennas. This approach is also useful in demonstrating concurrent MIMO operations and boosting channel capacity and throughput. Further research is being conducted on eight port antennas to improve other important parameters, such as gain, efficiency, compactness, and dual polarization for 5G and mm-wave applications.

## Figures and Tables

**Table 1:** Summary of the existing literature.

Ref.	No. of Ports	Isolation Techniques	Polarization	CMA Assisted	Band
[19]	4	Gap between radiation and ground plane	Linear	No	Wide
[20]	4	A cross-parasitic microstrip	Linear	No	Multi
[23]	2	Slot above the feedline	Circular	No	Narrow
[24]	2	A line patch introduced	Circular	No	Wide
[25]	2	Without the incorporation of decoupling unit	Circular	No	Wide
[26]	2	Two antennas are placed in the far field region	Circular	No	Narrow
[27]	2	Straight strip is attached to an L-shaped ground strip	Circular	No	Wide
[28]	2	Parasitic patch with the diagonal position of DRAs	Circular	No	Wide
[29]	2	----	Circular	No	Wide
[30]	2	Two identical DRAs with cross slot	Circular	No	Narrow
[31]	2	FSS superstrate	Circular	No	Narrow
[32]	3	----	Circular	No	Wide
[33]	4	Inserting λ/2 diagonal strips between the radiators	Circular	No	Wide
[34]	4	Cross-shaped incorporated into the ground plane	Circular	No	Wide
[35]	4	Metal strips with 180° out of phase	Circular	No	Wide
[36]	4	An L-shaped stub	Hybrid	Yes	Multi
[Prop. Work]	4	----	Circular	Yes	Wide

**Table 2:** CMA parameters of the proposed antenna.

Modes	Mode 1	Mode 2	Mode 3	Mode 4	Mode 5	Mode 6
**Resonant Modes**	🗴	🗸	🗴	🗸	🗸	🗸
**Frequencies (GHz)**	-	5.32	-	6.80	9.61	10.40
**Non-Resonating Modes**	🗸	🗴	🗸	🗴	🗴	🗴
**Orthogonal Modes**	Mode 2 and Mode 4		Mode 4 and Mode 5		Mode 5 and Mode 6	
**Frequencies (GHz)**	6.19		8.40		9.90	
**MS**	0.81		0.98		0.76	
**CA**	71.7°		73.3°		77.7°	

**Table 3:** Geometrical parameters of the proposed antenna.

Parameter	*W_s_*	*L_s_*	*W_p_*	*L_p_*	*W_p_*	*W_f_*	*g*
**Value (mm)**	25	25	18	18	6	2.75	0.4
**Parameter**	***a***	***b***	***c***	***d***	***e***	***r_in_***	***r_out_***
**Value (mm)**	8.3	8.5	8	1	4	2	3

**Table 4:** Comparative analysis of different antenna design stages.

Parameters	Ant @ 1	Ant @ 2	Ant @ 3
**Resonant frequency (GHz)**	5.41–10.54	5.56–11	5.59–11
**Impedance Bandwidth (%)**	64.3	>65.7	>65.2
**Cutoff-frequency (GHz)**	7.97	>8.28	>8.29
**Axial ratio (GHz)**	5.92–9.50	5.69–10.05	5.54–10.31
**Axial ratio bandwidth (%)**	46.4	55.4	60.1
**Cutoff-frequency (GHz)**	7.71	7.87	7.92
**Peak gain (dBi)**	3.66 @ 9.8 GHz	4.55 @ 8.8 GHz	5.48 @ 8.4 GHz

**Table 5:** Comparison of dual port results.

Results	Impedance Bandwidth			Axial ratio Bandwidth		
	%	GHz	*fc*	%	GHz	*fc*
**Simulated**	65.7	5.56–11 (above)	8.28	55.4	5.69–10.05	7.87
**Measured**	68.2	5.40–11 (above)	8.20	57.3	5.55–10.01	7.78

**Table 6:** Comparison of quad port results.

Results	Impedance Bandwidth			Axial Ratio Bandwidth		
	%	GHz	*fc*	%	GHz	*fc*
**Simulated**	65.2	5.59–11 (above)	8.29	60.1	5.54–10.31	7.925
**Measured**	68.8	5.37–11 (above)	8.30	58.6	5.61–10.26	7.935

**Table 7:** Comparison of MIMO antenna from the reported literature.

Ref.	Antenna Size w × l × h (mm)	Impedance Bandwidth			Axial Ratio Bandwidth			Isolation > dB	Peak Gain (dBi)
		%	GHz	*fc*	%	GHz	*fc*		
**[23]**	97 × 27.69 × 1.524	9.2	5.49–6.02	5.7	1.5	5.77–5.86	5.8	33	5.3
**[24]**	66 × 66 × 1.6	34.1	1.82–2.57	2.1	18.9	2.15–2.60	2.3	24	2.2
**[25]**	37 × 30 × 0.8	28.1	3.20–4.25	3.7	28.1	3.20–4.25	3.7	15	2.5
**[26]**	36.2 × 13.7 × 0.813	19.2	5.20–6.30	5.7	19.2	5.20–6.30	5.7	20	5.8
**[27]**	60 × 60 × 1.6	81.1	2.00–4.76	3.3	59.6	2.00–3.70	2.8	15	4.0
**[28]**	35 × 35 × --	34.3	3.50–4.95	4.2	20.5	3.58–4.40	3.9	28	6.2
**[29]**	80 × 80 × 5	36.7	5.71–8.20	6.9	45.5	7.72–8.04	7.9	15	3.8
**[30]**	126 × 76 × 0.8	9.2	2.36–2.59	2.4	4.8	2.39–2.51	2.4	20	5.6
**[32]**	48 × 29 × 1.6	14.5	5.40–6.25	5.8	1.5	5.61–5.70	5.6	18	4.7
**[33]**	60 × 60 × 1.6	63.8	3.20–6.20	4.7	18.9	4.30–5.20	4.7	25	3.0
**[34]**	70 × 68 × 1.6	105.8	4.0–13.0	8.5	67.7	4.20–8.50	6.3	18	6.4
**[35]**	45 × 45 × 1.6	143.9	2.20–13.5	7.8	52.4	3.80–6.50	5.15	18	6.8
**Prop.** **Work**	50 × 50 × 0.4	64.9	5.37–11.0	8.3	58.6	5.61–10.26	7.9	21	5.6

## Data Availability

Not applicable.
